# Whither Are We Drifting?

**Published:** 1893-03

**Authors:** 


### Editorial.

### WHITHER ARE WE DRIFTING?

THE changes which have been made in dentistry during the past forty years are well known to the profession at large, and need no reiteration here; but the effect produced may not be appreciated nor fully studied to comprehend whether change means advancement in the best sense, or whether it may not imply a retrograde in many directions. The question that heads this article, therefore, becomes of importance in that the duty is incumbent upon each individual to know whether, as a profession, we are drifting towards higher results in practice.

The age of machinery is upon us, with all its tendencies of good or ill, affecting all the relations of life, and has become an important factor in the changes which have so impressed the practice of dentistry in the period named.

The time antedating the introduction of cohesive gold was one of preparation. It was not an active period in the direction of the introduction of new appliances.

The necessity then existing of formulating methods of procedure was a paramount impulse, and held the thought of that day so closely that radical changes in instruments were not seriously considered. This could not last, and as years were added to years the inventive mind was led to a multiplication of devices, and labor-saving appliances so extended that it has become difficult for the average practitioner to understand their value or to make use of them in practice.

With the introduction of rubber as a dam, came, unfortunately, the loss of the knowledge how to use napkins. With its use were introduced measures to more effectually retain it in position. With cohesive gold came the requirement for means to accomplish more rapid work and for more perfect consolidation of this material, and the mallet of varied forms was generally recognized as essential. The slow hand-processes gave place to the drill used by power, and the dental engine was a fixture in nearly every office. The separating of teeth by older methods gave way in large degree to instruments of force. The motor has taken the place of the foot, and the end is not yet.

This brief statement, while not covering all the changes made, is sufficient to show that the tendency has been towards rapidity, without thought whether this would result in benefit to the patient, or, indeed, to the real advance of the profession.

It is a truism to say that every piece of machinery introduced has been followed by a loss and a gain,—a loss of the skill required to effect a given result without it, and a gain in a new development of skill in its use and facility in accomplishing more in a given period. This is, or should be, recognized. The balance between loss and gain means the amount of true progress made in any direction.

The question of greatest import to us is not whether, as dentists, we have been benefited physically or financially by the so-called improvements made, but whether our claim to be a body of workers in a branch of the healing art has been well sustained; or whether, in other words, our patients have been, upon the whole, benefited. This question is one too broad to be answered in full in the brief space allotted, but a few considerations may not be out of place to demonstrate, if possible, that change is not always progress, nor is rapidity always conducive to the well-being or ultimate good of those we are called upon to treat.

The older members of the profession of dentistry will doubtless agree with the writer that the period referred to—that antedating cohesive gold—was one in which lesions, now very familiar, were strikingly absent. While this in a degree may be attributed to a want of true pathological knowledge, it still remains a fact that the dividing-line between the old and the new dentistry brought with it a series of complications, if not unknown, at least very rare, and not constituting a disturbing element in practice.

The introduction of the dam, with the necessity of having it closely fitting the cervical border, involved irritation of delicate tissues. This was further aggravated by the ligature, and subsequently by the clamps introduced. What followed? The impression made by these upon the pericementum meant a local irritation and an introduction of a train of evils of important pathological significance. It would, perhaps, be assuming too much to state that pyorrhœa alveolaris has frequently been produced in this way, but the evidence is sufficient to show that its origin can thus be traced, and, in the judgment of the writer, is more often the cause than practitioners are willing to admit. It may be said in extenuation that, recognizing this to be true, it could only occur in careless hands. This is conceded, but in the hurry of operations it is questionable whether carelessness is not the rule rather than the exception. How many after employing these appliances take the trouble to use antiseptics at the margins of the gums? Perhaps not many, and yet, as a prophylactic measure, this is essential. How many, in forcing the clamp to place, take the trouble to examine whether this impinges on the pericementum? or how many regard with any concern the position of the ligature, often in place for an hour, and even longer? How many view with any anxiety the possible effect of a forceful separation of teeth by the instruments in general use? Do they take into consideration the anatomical structure of parts and the effect of their displacement? Would they in after-years be disposed to charge themselves with any subsequent malarrangement of the teeth? Not many, it is feared, are thus thoughtful; and yet all the contingencies named are among the possibilities, and these might be elaborated to a far greater extent.

The effect of the mallet is so well understood by every operator that it would seem useless, to expend many words upon it, yet it is questionable whether many stop in the hurry of practice to think of the physiological disturbances which may be produced by its use,—the shock to delicate organisms, the local effect upon the teeth, the injury to the material used, and the many more or less serious complications which may arise from the severe blows often repeated.

There is a proper and an improper use of instruments, and this applies with almost equal force to the simple as well as to the complex. Every one is valuable and necessary, but to use them correctly means physiological and pathological knowledge, and where this is possessed there need be no fear of injury, where conscience is in command.

We are drifting rapidly more and more into these professional conveniences, and, it is feared, with almost equal certainty into a careless disregard of that higher standard which means the greatest good attainable for the patient, with the least expenditure of physical and nervous energy for the operator.

